# Asymmetric dimethylarginine levels in patients with cutaneous anthrax: a laboratory analysis

**DOI:** 10.1186/1476-0711-13-12

**Published:** 2014-03-26

**Authors:** Mahmut Sunnetcioglu, Zafer Mengeloglu, Ali Irfan Baran, Mustafa Karahocagil, Mehmet Tosun, Abdulkadir Kucukbayrak, Mehmet Resat Ceylan, Hayrettin Akdeniz, Cenk Aypak

**Affiliations:** 1 Department of Infectious Diseases and Clinical Microbiology, Yuzuncu Yil University, Faculty of Medicine, Van, Turkey; 2 Department of Medical Microbiology, Abant Izzet Baysal University, Faculty of Medicine, Bolu, Turkey; 3 Department of Medical Biochemistry, Abant Izzet Baysal University, Faculty of Medicine, Bolu, Turkey; 4 Department of Infectious Diseases and Clinical Microbiology, Abant Izzet Baysal University, Faculty of Medicine, Bolu, Turkey; 5 Department of Family Medicine, Diskapi Yildirim Beyazit Training and Research Hospital, Ankara 06110, Turkey

**Keywords:** Dimethylarginine, Infection, Anthrax, Vasculitis

## Abstract

**Background:**

Asymmetric dimethylarginine (ADMA), the main endogenous inhibitor of nitric oxide synthase, is considered to be associated with endothelial dysfunction. High ADMA levels have been shown to be related with disorders causing vascular inflammation such as hypertension, hypercholesterolemia, atherosclerosis, chronic heart failure, stroke and sepsis. Cutaneous anthrax (CA) is a serious infectious disease which may cause vasculitis. The aim of the study was to investigate the serum ADMA levels in patients with CA.

**Methods:**

A total of 35 serum samples of the patients with CA and 18 control sera were tested for ADMA levels using ADMA ELISA kit (Immunodiagnostik AG, Bensheim, Germany).

**Results:**

ADMA levels were found to be significantly higher in the patients group than the controls (p < 0.001). In addition, ADMA levels were found to be positively associated with sedimentation rates (R = 0.413; p = 0.026), and inversely associated with international normalized ratio (INR) levels (R = -0.46; p = 0.011). A cut-off value of 0.475 of ADMA had a sensitivity of 74.3%, specificity of 77.8%, and accuracy of 75.5% in the diagnosis of CA.

**Conclusion:**

Although the exact mechanism still remains unclear, ADMA levels could be related to immune activation in CA. In addition, these data might suggest the higher ADMA levels in patients could be due to the perivascular inflammation and vasculitis in CA.

## Background

Nitric oxide (NO) is reported to be an important mediator of vascular tone. Asymmetric dimethylarginine (ADMA) has been shown to be the main endogenous inhibitor of NO synthase and it regulates NO formation. High ADMA levels have been shown to be related to disorders causing vascular inflammation, such as hypertension, hypercholesterolemia, atherosclerosis, chronic heart failure, stroke, and sepsis. Therefore, ADMA is considered to be associated with endothelial dysfunction. Furthermore, ADMA is considered to be a predictive marker of mortality in critically ill patients [1-4].

Anthrax is a rare, potentially fatal zoonotic disease caused by the bacterium Bacillus anthracis, which can infect both animals and humans [5]. Infection via inhalation of Bacillus anthracis spores can result in a mortality rate of up to 96% [5,6]. Cutaneous anthrax (CA), the most common form of the disease, accounts for 95% of all anthrax cases [7]. It is acquired when spores enter through a cut in the skin; it is characterized by the formation of a black scar surrounded by prominent edema and vesicles, and it is reported to cause vasculitis [6]. Bacteremia and toxemia following cutaneous infection can lead a fatality rate ranging from 20% to 25% among untreated cases [6,7].

This study aimed to evaluate the serum ADMA levels in patients with CA.

## Material and methods

### Sera

In total, sera samples from 35 patients with accurate diagnosis of CA were included in the study. Of these, five sera samples were collected from patients admitted into the clinics at Abant Izzet Baysal University (AIBU) Faculty of Medicine, and the remaining 30 sera samples were collected from patients at Yuzuncu Yil University Faculty of Medicine. Eighteen healthy subjects without a history of chronic or recurrent disease served as the controls.

The study protocol was approved by the local ethics committee. All the subjects were informed about the study, and written consent was obtained from each subject.

### Testing for ADMA levels

The symmetric dimethylarginine levels were tested using an ADMA ELISA kit (Immunodiagnostik AG, Bensheim, Germany) via the ELISA method in the Department of Medical Biochemistry at the AIBU Faculty of Medicine.

### Statistical analysis

The continuous variables were tested for normality using the Shapiro-Wilk test. The normally distributed values were presented as mean values (± standard deviation); otherwise, they were presented as median values (the interquartile range). A Chi-square test was used for intergroup comparisons. An independent sample *T* test or a Mann–Whitney *U* test was used for comparison between the two groups. Spearman’s rank correlation test or Pearson correlation test was used for the correlation and relationship between the indicated parameters. The serum ADMA level’s capacity to predict the presence of the disease in patients was analyzed using receiver operating characteristic (ROC) curve analysis. The sensitivity and specificity were presented when a significant cut-off value was observed. A p value of less than 0.05 was considered to be statistically significant.

## Results

**Figure 1:**
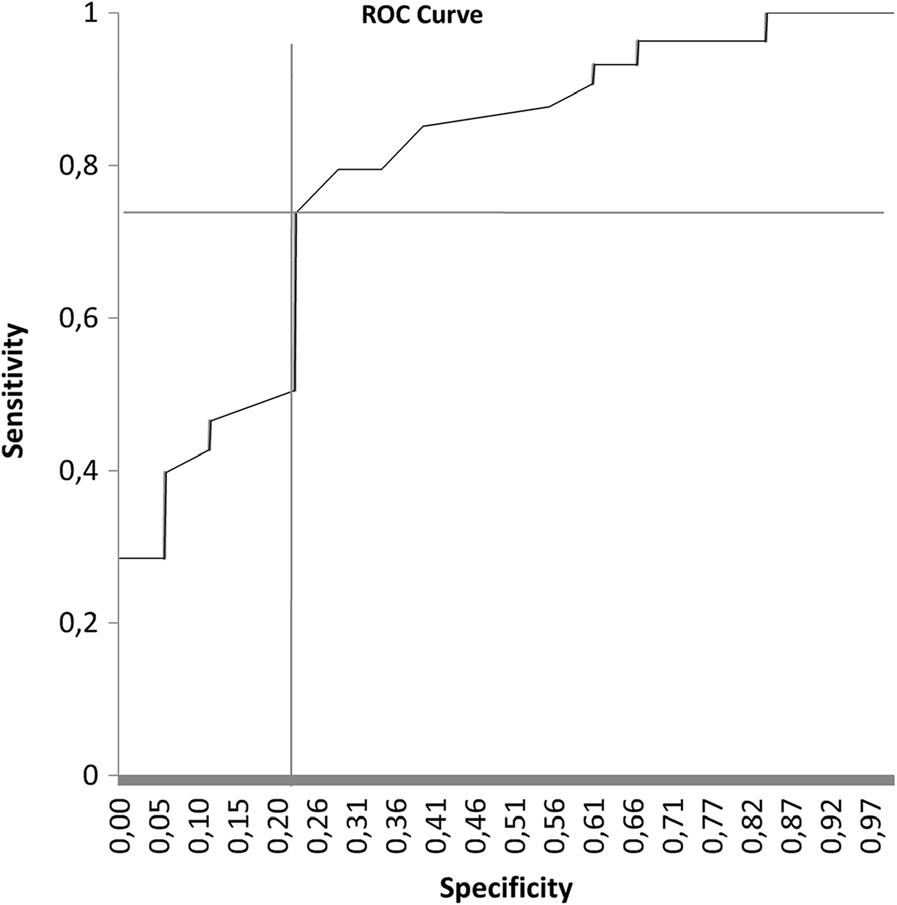
In the ROC analysis performed to predict ADMA levels in anthrax patients, a cut-off value of 0.475 had a sensitivity of 74.3%, specificity of 77.8%, positive predictive value of 86.7%, negative predictive value of 60.9%, and accuracy of 75.5 (AUC: 0.801; p < 0.001; LB:0.675; UB: 0.927; in confidence interval of 95%).

No significant differences were found between the groups in terms of age and gender. The ADMA levels were found to be significantly higher in patients with CA than they were in the healthy controls (p < 0.001) (Table 1). In the correlation analysis, the ADMA levels were found to be positively associated with sedimentation rates (R = 0.413; p = 0.026), and they were found to be inversely associated with international normalized ratio (INR) levels (R = -0.46; p = 0.011). No association was found between ADMA and the rest of the other laboratory variables (Table 2). In the ROC analysis performed to predict the ADMA levels in patients, a cut-off value of 0.475 had a sensitivity of 74.3%, specificity of 77.8%, positive predictive value of 86.7%, negative predictive value of 60.9%, and accuracy of 75.5 (AUC: 0.801; p < 0.001; LB:0.675; UB: 0.927; CI 95%) (Figure 1).

**Table 1:** Differences between the groups according to age, gender and ADMA levels

**Variables**	**Control group**	**Patient group**	***p***
Age (Mean ± SD)	35.76 ± 18.43	37.32 ± 21.69	0.41
Gender (Male/Female)	9/9	13/22	0.37
ADMA level [Median (IQR)]	0.44 (0.13)	0.59 (0.26)	<0.001

SD: Standard deviation; IQR: Interquartile range.

**Table 2:** The correlations between different variables in the patients group

**Variables**	**ADMA**		**CRP**		**Sedimentation**		**WBC**	
	r	p	r	p	r	p	r	p
Age	-0.096	0.59	-0.15	0.43	0.307	0.11	-0.222	0.22
ADMA								
CRP	0.24	0.2						
Sedimentation	0.413	0.026	0.28	0.14				
WBC	0.076	0.68	0.289	0.12	0.008	0.97		
ALT	-0.049	0.79	0.156	0.4	0.086	0.66	0.025	0.89
AST	0.172	0.34	0.286	0.11	-0.18	0.35	0.268	0.14
Albumin	0.092	0.64	-0.016	0.94	0.097	0.65	0.08	0.69
Glucose	0.052	0.78	-0.215	0.25	0.068	0.72	0.057	0.76
Urea	0.144	0.46	0.37	0.06	0.582	0.002	0.319	0.1
Creatinine	-0.003	0.99	0.035	0.85	0.296	0.2	0.241	0.18
PT	-0.33	0.08	0.17	0.38	0.255	0.2	0.238	0.21
INR	-0.46	0.011	0.291	0.13	-0.09	0.66	0.18	0.34

ADMA: Asymmetric dimethylarginine CRP: C-reactive protein WBC: White blood cell AST: Aspartat amino transferase ALT: Alanin amino transferase PT: Prothrombin time INR: International normalized ratio.

## Discussion

ADMA, a non-selective strong inhibitor of NO synthase, is accepted to be a biomarker of endothelium dysfunction [8]. ADMA was shown to be increased in hypercholesterolemia, hyperhomocysteinemia, hypertension, diabetes mellitus, insulin resistance, chronic heart failure, hyperthyroidism, hemorrhagic shock, preeclamptic pregnancy, multi-organ failure, and sepsis [8-13]. Those results indicate that the synthesis and release of ADMA could be increasing during inflammation. Moreover, it was considered that NO and ADMA play a role in the pathogenesis of many cutaneous diseases [1,7,14-16]. Rowe et al. showed increased NO synthase expression levels in patients with atopic dermatitis, allergic dermatitis, and psoriasis [1]. Sahin et al. found both ADMA and NO levels to be significantly higher in patients with Behcet’s disease [15]. In addition, increased NO was reported to be associated with other inflammatory diseases, such as rheumatoid arthritis, systemic lupus erythematosus, Sjögren’s syndrome, vasculitis, and osteoarthritis [15].

Although its incidence in developed countries has been very low owing to improvements in animal husbandry and handling of animal products, anthrax captured attention after October 2001 because of bioterrorism attack through the United States postal system and the subsequent identification of anthrax in 22 patients, including 11 with cutaneous disease [6,17,18]. CA is still endemic in the eastern and southeastern regions of Turkey due to the presence of uncontrolled livestock [14]. CA frequently occurs with direct contact with infected animals through a skin cut or an abrasion. The infection was transmitted to all of our patients in the same way.

In the present study, the ADMA levels were found to be significantly higher in the CA patients than in the controls, which is consistent with other inflammatory skin disorders. Although the exact mechanism underlying the increased ADMA levels in CA patients is not clear, this increase could be due to perivascular inflammation and vasculitis in CA, which was previously reported [6]. Shieh et al. found vasculitis and various degrees of inflammation were present in the lower epidermis and dermis of CA case specimens. Warfel et al. also showed endothelial dysfunction in anthrax [16]. Unfortunately we did not obtain skin biopsy samples of our cases.

ADMA levels were also found to be high in patients with HIV and it was stated that increased ADMA production might be related to increased activity in the immune system [19]. In addition, a decrease in ADMA levels was shown in HIV patients undergoing antiretroviral therapy [20]. The positive association between ADMA and sedimentation rate, which was shown in our study, is consistent with the hypothesis of increased ADMA levels and could be related to immune activation.

Although to our knowledge, this is the first study to investigate serum ADMA enzyme activity in patients with CA, it has limitations. First, our study design has not allowed us to investigate if ADMA activity is uniquely associated with CA. May be, a third group of sick patients, with elevated inflammatory biomarkers, but without increased ADMA levels, could have been included to highlight the issue ideally. Second, as it was mentioned previously we did not obtain skin biopsies to evaluate the histopathologic changes including perivascular inflammation. However, in the current study, we observed that serum ADMA levels were significantly higher in patients with CA than in the healthy controls.

In conclusion, our results suggest that ADMA contributes to the inflammation which is seen in CA. Further studies are needed in order to highlight the role that ADMA plays in the mechanism and the pathogenesis of CA.

## Competing interest

The authors declare that they have no competing interests.

## Authors’ contributions

MS, MK, ZM, and HA designed the study; MS, ZM, MK, AIB, AK, MRC, HA, and MT collected the data; ZM and MT analyzed the data; and MS, MK, ZM, AIB, AK, MRC, MT, HA, and CA drafted the manuscript and revised it to insure that it contained important intellectual content. All the authors read and approved the final manuscript.
